# Medication Management Service for Old Age Homes in Hong Kong Using Information Technology, Automation Technology, and the Internet of Things: Pre-Post Interventional Study

**DOI:** 10.2196/24280

**Published:** 2021-02-10

**Authors:** Kei Hong So, Cheuk Wun Ting, Chui Ping Lee, Teddy Tai-Ning Lam, Sau Chu Chiang, Yin Ting Cheung

**Affiliations:** 1 School of Pharmacy Faculty of Medicine The Chinese University of Hong Kong Hong Kong Hong Kong (China); 2 Hong Kong Pharmaceutical Care Foundation Hong Kong Hong Kong (China)

**Keywords:** medication management, old age homes, information technology, automation, Internet of Things

## Abstract

**Background:**

Innovation in technology and automation has been increasingly used to improve conventional medication management processes. In Hong Kong, the current practices of medication management in old age homes (OAHs) are time consuming, labor intensive, and error prone. To address this problem, we initiated an integrated medication management service combining information technology, automation technology, and the Internet of Things in a cluster network of OAHs.

**Objective:**

This pilot study aimed to evaluate the impact of the medication management program on (1) medication management efficiency, (2) medication safety, and (3) drug wastage in OAHs. We compared the time efficiency and the reductions in medication errors and medication wastage in OAHs before and at least 2 weeks after the implementation of the program.

**Methods:**

From November 2019 to February 2020, we recruited 2 OAHs (serving 178 residents) in Hong Kong into the prospective, pre-post interventional study. The interventional program consisted of electronic medication profiles, automated packaging, and electronic records of medication administration. Using 3-way analysis of variance, we compared the number of doses prepared and checked in 10-minute blocks before and after implementation. We received anonymous reports of medication errors from OAH staff and analyzed the results with the Fisher exact test. We also calculated the quantity and cost of wasted medications from drug disposal reports.

**Results:**

The number of doses prepared and checked in 10-minute blocks significantly increased postimplementation (pre: 41.3, SD 31.8; post: 70.6, SD 22.8; *P*<.001). There was also a significant reduction in medication errors (pre: 10/9504 doses, 0.1%; post: 0/5731 doses; *P*=.02). The total costs of wasted medications during January 2020 in OAH 1 (77 residents) and OAH 2 (101 residents) were HK $2566.03 (US $328.98) and HK $5249.48 (US $673.01), respectively.

**Conclusions:**

Our pilot study suggested that an innovative medication management program with information technology, automation technology, and Internet of Things components improved the time efficiency of medication preparation and medication safety for OAHs. It is a promising solution to address the current limitations in medication management in OAHs in Hong Kong.

## Introduction

Reports from the literature have shown that medication errors in long-term care facilities are common and can have deleterious consequences. One study of 865 long-term care facilities in Japan found that the incidence rate of medication errors was 40.0 per 1000 residents [1]. A local study in Hong Kong reported that 46.4% of drug-related problems in old age homes (OAHs) were drug administration errors [2]. To address this problem, user-friendly information technology (IT) systems and electronic medication administration records (eMARs) have been increasingly used in long-term care facilities as they allow more reliable input and more rapid retrieval of medical information [3-5]. A systematic review summarized that adopting IT in healthcare services could successfully decrease medication errors, increase adherence to guidelines, and enhance surveillance [6]. While some less recent studies detected medication errors still existing after using automated dispensing system, Beobide Telleria et al found a reduction of 91% in dispensing errors after using an automated packaging system in seven nursing homes in Spain [3,7,8]. The use of automated packaging and dispensing systems has effectively improved safety in the dispensing and administration of solid drugs in nursing homes.

In Hong Kong, people 65 years or older constituted 15.9% of the total population in 2016. Among people in this age group, 8.1% were living in non-domestic households such as one of the 734 licensed OAHs [9,10]. Improving the drug distribution model in OAHs is one of the top priorities in Hong Kong as it is often associated with medication wastage. In most cases, OAH residents have regular medical follow-ups from the hospitals and clinics of the Hospital Authority (HA), which is part of the public health care sector [2]. As the time between each follow-up visit is long [11], 6-month to 1-year supplies of medications are typically dispensed at a single setting to ensure that there is sufficient medication to last until the next follow-up visit. However, as older patients may experience frequent symptom changes that lead to subsequent adjustment or early discontinuation of medications, a large quantity of surplus medication is wasted [11]. One study calculated that the total extrapolated annual cost of medications requiring disposal in all OAHs in Hong Kong was over HK $5,800,000 (US $743,589), including more than 10 million units of oral solid preparations that were estimated to be discarded [11].

Other than being associated with high wastage, the conventional medication preparation process is also labor intensive, time consuming, and error prone. In Hong Kong, OAH nursing staff manually prepare and check all medications, and all medication management activities (medication preparation, checking, and administration) in OAHs are made with reference to and recorded on their in-house paper medication administration records (MARs). This laborious process can result in medication errors and compromise patient safety. In a local survey, 36.8% of the OAHs reported that there was at least one incident of incorrect drug administration over the past 5 years [12]. There were 126 officially reported cases of suspected medication incidents in OAHs from 2008 to 2010 [13].

To address the limitations of the current medication management process in OAHs, the Hong Kong Pharmaceutical Care Foundation Ltd (HKPCF) [14], a local nongovernmental organization, initiated the Integrated Old Age Home Medication Management Program (“the Program”) in 2019. Funded by a philanthropic foundation, the Program aims to utilize various technological systems to provide an integrated medication management service to a cluster network of OAHs. This initiative is innovative and, to our knowledge, the first of its kind in Hong Kong.

To date, few studies have systematically evaluated both the efficiency and safety of such comprehensive medication management services. Therefore, we carried out a pilot study to evaluate the impact of the Program on (1) medication management efficiency, (2) medication safety, and (3) drug wastage in OAHs.

In this paper, we described the components of the medication management program and compared the time efficiency and the reductions in medication errors and medication wastage in OAHs before and at least 2 weeks after the implementation of the program. Based on this pilot study, we discussed potential limitations of the program and highlighted directions for future research.

## Methods

### Design and Study Population

**Figure 1:**
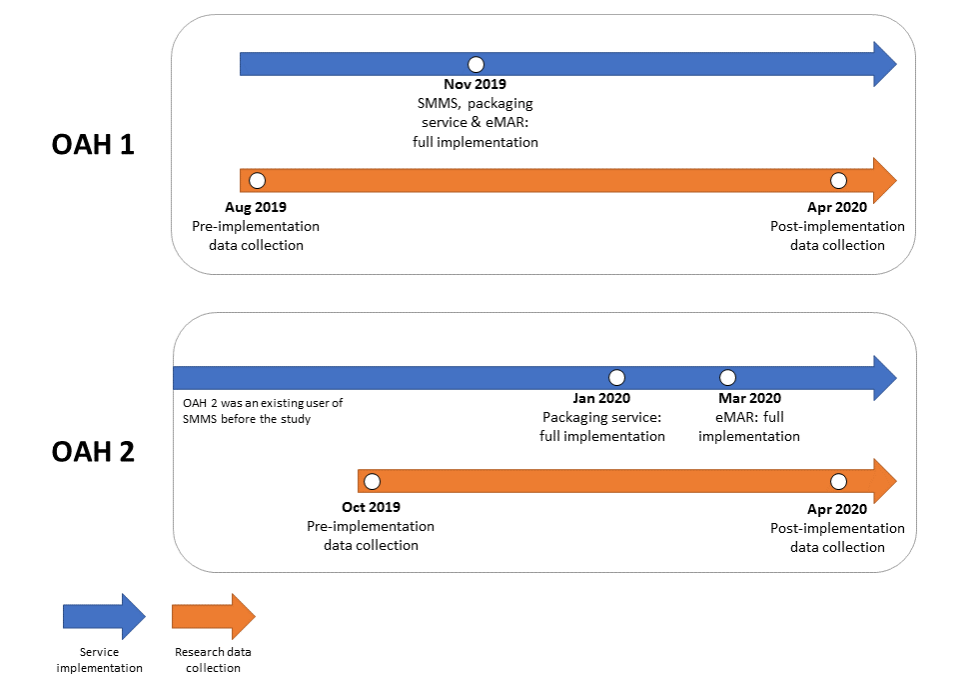
Timeline of implementation of the Integrated Old Age Home Medication Management Program and study data collection of the two Old Age Homes recruited. eMAR: electronic medication administration record; OAH: old age home; SMMS: SafeMed Medication Management System.

The pilot study was a prospective and interventional study that included preintervention and postintervention evaluation of efficiency outcomes and medication error rates. Time-motion methods [15] were used to quantify the number of doses prepared and checked during the medication handling processes. Recruitment of OAHs started in August 2019, and pilot data collection ended in April 2020. Figure 1 summarizes the timeline of Program implementation and data collection for the 2 OAHs that we recruited for this pilot study.

Approval was obtained from the Survey and Behavioural Research Ethics Committee of the Chinese University of Hong Kong (reference number: SBRE-19-106) (Text S1 in Multimedia Appendix 1). We obtained verbal consent from the staff members observed in the OAHs and the HKPCF prior to the data collection period.

### Intervention

The Program consisted of three components combining elements of IT, automation technology (AT), and the Internet of Things (IoT): (1) electronic medication profiles in the SafeMed Medication Management System (SMMS); (2) a centralized automation-assisted multidose packaging service using the Automated Tablet Dispensing and Packaging System (ATDPS); and (3) an eMAR app on mobile tablet devices. SMMS is an electronic medication management system developed by pharmacists at HKPCF for maintaining updated medication profiles of OAH residents with the support of a comprehensive drug database. ATDPS consists of an automated packaging machine that generates multidose drug pouches containing tablets or capsules to be administered to individual residents during different medication rounds. Automated multidose packaging is done with ATDPS such that medications of a patient at a particular time of administration are packaged in the same drug pouch. Automated packaging, instead of manual packaging, is adopted to diminish human errors under the fatigue of repetitive procedures of dispensing and packaging, hence improving medication safety. All beneficiary OAHs received all three components as the intervention except existing users of the SMMS, for which only (2) and (3) were provided in addition to the SMMS as the intervention.

**Figure 2:**
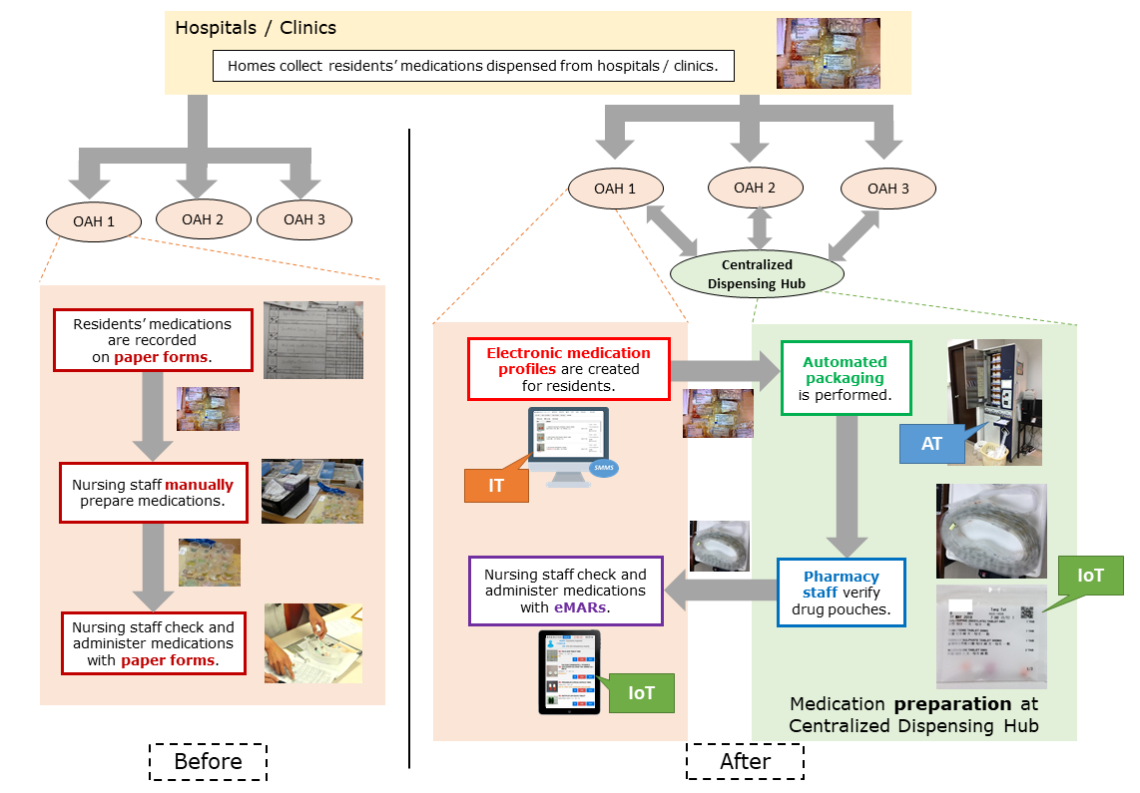
Medication management process before and after implementation of the Integrated Old Age Home Medication Management Program. AT: automation technology; eMARs: electronic medication administration records; IoT: Internet of Things; IT: information technology; OAH: old age home.

Figure 2 summarizes the medication management workflow before and after the implementation of the Program. Before implementation of the Program (left), dispensed medications collected from hospitals and clinics are recorded, prepared, checked, and administered manually by OAH nursing staff. After implementation (right), nursing staff create medication information electronically using SMMS. The medications are collected by staff of the centralized dispensing hub for automated packaging and verification. Packaged medications are sent back to OAHs for checking and administration.

Throughout the process of implementing the intervention, HKPCF pharmacists provided training, regular follow-up, and technical assistance for OAH nursing staff to familiarize themselves with the use of the new system. In the postimplementation phase, “medication preparation” of daily medications (including automation-assisted packaging and additional verification of packaging errors) was conducted by HKPCF in a centralized medication management hub (“the hub”). Medications packaged in drug pouches were delivered to the OAHs in batches of 2 to 3 days of medication supply. Paper MARs were replaced by the eMAR app, which possesses features such as bar-coded patient identification and electronic signatures, for “medication checking” and “medication administration.” Figure S1 in Multimedia Appendix 1 summarizes the flow of medications and information, and Text S2 in Multimedia Appendix 1 describes in detail the workflow and components of the Program.

### Primary Outcome: Time Efficiency

The primary outcome of the study was the time efficiency of medication preparation and checking, represented by the mean number of doses prepared and checked in 10-minute blocks. The time-motion approach, which has been widely adopted to evaluate the efficiency of workflow, was used to observe the processes of medication preparation and checking.

During data collection, all staff members performed their duties as usual, and an investigator or a helper of the research team videotaped the processes. An investigator viewed the videos and calculated the number of doses prepared or checked in 10-minute blocks in the preimplementation and postimplementation phases. Time during which staff members were interrupted during the medication management processes was excluded. The postimplementation data were collected after at least 2 weeks of run-in time of full service implementation.

### Secondary Outcomes: Medication Safety and Medication Wastage

The secondary outcomes were the number of medication errors and medication wastage. We evaluated the impact on medication safety by measuring the number of medication errors captured within 5 to 10 days during both the preimplementation and postimplementation phases. In the postimplementation phase, we collected data at least 2 weeks after the full implementation of the Program.

The OAH nursing staff voluntarily reported all medication errors identified during medication checking and administration using an anonymous incident-reporting form in Chinese, modified from the Medication Risk Management Report in *Guidelines on Drug Management in Residential Care Homes 2018* [16]. Errors were calculated as the proportion of problematic doses over the total number of doses processed during the period of data collection from the SMMS.

We retrieved data on medication wastage in January 2020 from the drug disposal reports in the SMMS, which estimated the number of dispensed drugs that were not administered to the patient due to early discontinuation of treatment. Under the centralized automated packaging service of the Program, any unused oral solid medications (pills, tablets, capsules, sustained-release tablets, etc) that are originally dispensed for one patient would be repackaged and supplied to another patient that requires the same medication. Hence, we made a reasonable assumption that wastage could be almost completely eliminated when medications were centralized for redistribution to all residents and that the calculated cost would reflect the cost of wasted medication that could potentially be avoided after the implementation of the Program. In the drug disposal reports, medications were classified according to dosage forms and therapeutic classes in the British National Formulary (BNF) [17]. Based on residents’ prescriptions and the unit prices of medications derived from HA data, the quantity and cost of wasted medications in the OAHs were calculated to quantify the scale of medication wastage.

### Data Processing and Statistical Analysis

We used SPSS, version 24 (IBM Corp), as the software for statistical analysis. We summarized the baseline characteristics of each OAH with descriptive statistics. We compared the number of doses prepared and checked in 10-minute blocks in the 2 recruited OAHs before and after the implementation of the intervention using 3-way analysis of variance (ANOVA). By calculating the standard deviation, we observed the variations within samples for medication preparation and checking for each OAH before and after implementation. To detect any significant changes in the number of medication errors, we conducted the Fisher exact test based on the number of correct and incorrect doses before and after implementation. We defined a statistically significant difference as a *P* value of less than .05.

## Results

### Characteristics of OAHs

For this pilot study, we recruited 2 out of the 8 OAHs participating in the Program. Table 1 shows the baseline characteristics of the 2 recruited OAHs (referred as OAH 1 and OAH 2). The age of residents, number of staff members, staffing ratios, and number of medications per resident were similar in both OAHs. OAH 1 was a new user of the SMMS, whereas OAH 2 was an existing user of the SMMS at the time of enrolment into the Program.

**Table 1:** Baseline characteristics of OAHs.

Characteristics			OAH^a^ 1		OAH 2
Care level^b^ [18]			Care and attention home for the elderly		Care and attention home for the elderly
Mode of operation^c^ [18]			Subvented home		Subvented home
District			Sham Shui Po		Kowloon City
Residents, n			77		101
**Age of residents (years)**					
	Mean (SD)	87.2 (6.9)		86.4 (8.9)	
	Median (IQR)	88 (8)		87 (12)	
	Range	67-104		67-105	
**Gender of residents, n (%)**					
	Male	18 (23.4)		36 (35.6)	
	Female	59 (76.6)		65 (64.4)	
**Staff members, n** [18]					
	Total staff members^d^	52.3		52.75	
	Nurses	9		7	
	Health workers	2		6	
Staff-to-resident ratio			1:1.5		1:1.9
Nursing staff–to-resident ratio^e^			1:7		1:7.8
**Oral medications per resident, n**					
	Mean (SD)	8.3 (3.6)		9.8 (3.7)	
	Median (IQR)	8 (5)		10 (4)	
	Range	0-21		1-21	
Status of using SMMS^f^			New user		Existing user

^a^OAH: old age home.

^b^Based on care level, OAHs in Hong Kong were classified into Homes for the Elderly (for older people with no or mild impairment), Care and Attention Homes for the Elderly (for older people with moderate impairment), and Nursing Homes (for older people with severe impairment).

^c^Based on mode of operation, OAHs in Hong Kong were classified into Subvented Homes, Contract Homes, Non-profit-making Self-financing Homes, and Private Homes.

^d^The number of staff members may not be an integer because some staff members were part-time employees.

^e^Nursing staff refers to nurses and health workers.

^f^SMMS: SafeMed Medication Management System.

### Primary Outcome: Time Efficiency

The mean number of doses prepared in 10-minute blocks for OAH 1 increased from 13.1 (SD 3.7) in the preimplementation phase to 77.1 (SD 9.1) in the postimplementation phase, while for OAH 2, there was an increase from 35.3 (SD 7.7) to 84.8 (SD 15.3) doses. The mean number of doses checked in 10-minute blocks in RCHE 1 increased from 24.7 (SD 12.6) to 67.2 (SD 13.0), but in OAH 2, it decreased from 92.0 (SD 7.1) to 53.1 (SD 33.7) (Table S1 in Multimedia Appendix 1). In other words, the time required to prepare 1000 doses of medications had dropped from 560.6 (SD 320.1) minutes to 126.3 (SD 18.5) minutes (Table S2 in Multimedia Appendix 1).

**Figure 3:**
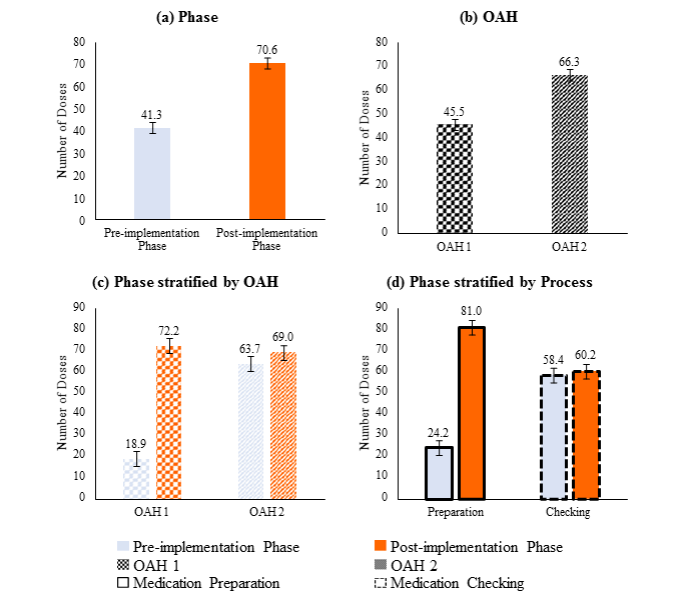
Three-way analysis of variance results (considering the effect of one or two factors). OAH: old age home.

**Figure 4:**
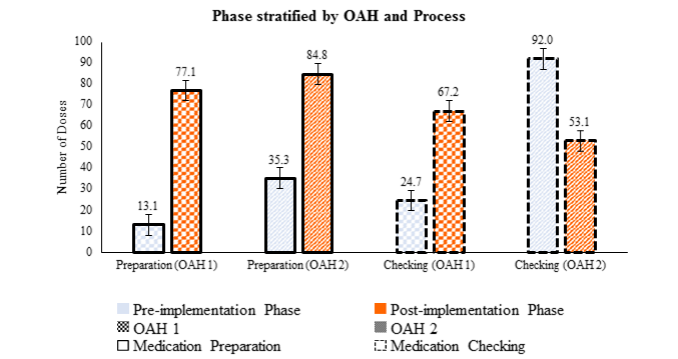
Three-way analysis of variance results (considering the effect of three factors). OAH: old age home.

The results of a 3-way ANOVA (Figures 3 and 4; Tables S3 and S4 in Multimedia Appendix 1) showed the mean number of doses processed in 10 minutes (error bars denote standard error), stratified by (a) preimplementation versus post implementation, (b) OAH 1 versus OAH 2, (c) pre- and post- comparison stratified by OAH, (d) pre- and post- comparison stratified by process (medication preparation/checking), and (e) pre- and post- comparison stratified by both OAH and process. Each of the main effect or interaction terms presented is statistically significant (*P*<.001) in the 3-way ANOVA analysis. Results showed a significant increase (*P*<.001) in the number of doses prepared and checked in 10-minute blocks after the implementation of the Program. These numbers also increased significantly (*P*<.001) in OAH 1 alone during the postimplementation phase. However, fewer doses were processed during checking in OAH 2 postimplementation.

### Secondary Outcome: Medication Errors

Due to time constraints and the expressed preference of OAH 1, we only collected the medication error data from OAH 2. During the data collection period in the preimplementation phase, a total of 9504 doses were handled, among which the nursing staff reported medication errors for 10 doses during medication checking and none during medication administration (Table 2). We classify the errors according to the nature of the errors and the medications involved in Tables S5 and S6 of Multimedia Appendix 1. We emphasize that the OAH nursing staff corrected all of these errors before medication administration, so they should be considered as “near misses” and did not result in actual errors.

During the data collection period in the postimplementation phase, there were a total of 5731 doses, among which nursing staff reported no medication errors during medication checking and medication administration (Table 2). Comparing the results before and after the intervention, we found a significant reduction (*P*=.02) in the number of medication errors using the Fisher exact test.

**Table 2:** Number of wrong and correct doses in preimplementation and postimplementation phases.

Phase	Doses in OAH^a^ 2, n		
	Wrong	Correct	Total
Preimplementation phase	10	9494	9504
Postimplementation phase	0	5731	5731

^a^OAH: old age home.

### Secondary Outcome: Medication Wastage

In January 2020, the total costs of wasted medications in OAH 1 (77 residents) and OAH 2 (101 residents) were HK $2566.03 (US $328.98) and HK $5249.48 (US $673.01), respectively. We estimated that the annual costs of wasted medications in OAH 1 and OAH 2 were HK $30,792.36 (US $3947.74) and HK $62,993.76 (US $8076.12), respectively, which were equivalent to HK $399.90 (US $51.27) per resident in OAH 1 and HK $623.70 (US $79.96) per resident in OAH 2.

Based on the dosage forms (Table 3), 3199 and 6762 tablets and capsules were wasted in OAH 1 and OAH 2, respectively, in January 2020. In terms of costs, the greatest proportion of wasted medications in OAH 2 was oral solid medications, constituting 52.8% (HK $2769.41 or US $355.05) of wasted medications. Oral solid medications also contributed to 27.3% (HK $701.51 or US $89.94) of wasted medications in OAH 1.

Based on the therapeutic classes in the BNF (Table S7 in Multimedia Appendix 1), drugs for central nervous system disorders made up the drug class with the highest cost of wasted medications in OAH 1 (HK $1328.24 or US $170.29; 51.8% of the total cost). In OAH 2, the group with the highest cost of wasted medication was drugs for nutrition and blood disorders (HK $1613.70 or US$ 206.88; 30.7% of the total cost).

**Table 3:** Medication wastage based on dosage forms.

Dosage form (unit)	OAH^a^ 1			OAH 2		
	n	Cost, HK $ (US $^b^)	n		Cost, HK $ (US $^b^)	
Oral solid medications (tablets/capsules)	3199	701.51 (89.94)	6762		2769.41 (355.05)	
Oral liquid medications (mL)	4355	158.13 (20.27)	3625		207.78 (26.64)	
Inhaled medications (puffs)	1385	279.63 (35.85)	1076		110.94 (14.22)	
Topical medications (grams)	0	0 (0.00)	0		0 (0.00)	
Transdermal patches (pieces)	62	1013.95 (129.99)	0		0 (0.00)	
Suppositories (suppositories)	72	255.71 (32.78)	65		230.85 (29.60)	
Eye drops (drops)	342	75.19 (9.64)	1142		325.9 (41.78)	
Sprays (doses)	208	64.35 (8.25)	0		0 (0.00)	
Mouthwashes (mL)	300	7.48 (0.96)	0		0 (0.00)	
Insulin injections (insulin units)	0	0 (0.00)	3004		278.05 (35.65)	
Other parenteral injections (μg)	3000	10.08 (1.29)	6240		1326.55 (170.07)	
Total	N/A^c^	2566.03 (328.98)	N/A		5249.48 (673.01)	

^a^OAH: old age home.

^b^US $1=HK $7.8.

^c^N/A: not applicable.

## Discussion

### Principal Findings

To the best of our knowledge, this is one of the first studies to examine the efficiency and safety of a medication management service integrating the use of IT, AT, and the IoT through three components: electronic medication profiles, centralized automated multidose packaging, and eMARs. Our study found that medications were more efficiently prepared in the hub and more efficiently checked in the OAHs after implementation of the Program.

The use of technology can drastically improve the workflow of medication management in OAHs. In a paper-based workflow, OAH staff need to flip through the paper MARs and read the instructions on the medication labels one by one during medication preparation and checking. They also need to manually take out the medication bags one by one and dispense them into suitable containers during medication preparation. With the implementation of eMARs, nursing staff can check the packaged pouches using a dedicated electronic device app showing the patient’s medication regimen, with authentic photographs to help them identify the correct medications. The data from the residents’ electronic profiles are rapidly sent from SMMS to ATDPS, which can package up to 60 drug pouches per minute [19]. Therefore, the IT- and AT-assisted workflow is undeniably more efficient than the traditional paper-based and manual workflow, as shown by the increased number of doses checked postimplementation in OAH 1. However, we note that the findings for OAH 2 showed otherwise; this may be due to the insufficient run-in time (2 weeks in OAH 2 versus more than 1 month in OAH 1), leading to reduced efficiency during the initial implementation phase of the Program. One comprehensive systematic review on the effect of health information technology on quality and efficiency of health care highlighted that data on time use and efficiency metrics were inconsistent [6]. The impact of automation on improving time efficiency requires long-term prospective evaluation.

The traditional mode of operation for medication management in OAHs puts a disproportionately heavy burden on nursing home staff. In Hong Kong, there is neither a statutory nor an administrative requirement to have any pharmacists or dispensers (pharmacy technicians) in OAHs [20]. To save costs, Health Workers (registered non-nurse OAH employees who have attended an approved short training course) [20] are frequently assigned as the main personnel for handling medications despite their inadequate training for this responsibility. Therefore, nursing staff often spend considerable effort and time spotting and preventing medication errors. After the implementation of the Program, the responsibilities for medication preparation and verification were transferred to pharmacists and dispensers in the hub. They are professionally trained in the handling of medications, and their expertise may have contributed to the improvement in efficiency, as well as the reduction in medication errors.

The Program also enables more effective use of manpower in OAHs for direct patient care services. Conventional time-consuming medication management activities place a huge burden on nursing staff members, who are also expected to provide other care services for residents. In a study in Australia, medication preparation and administration constituted approximately one-quarter of nurses’ 8-hour shifts in OAHs [21]. Upon implementation of the Program, the nursing staff in this study were completely relieved of their medication preparation duties. Therefore, the Program helped the OAH nursing staff to spare more time for the provision of higher-quality, patient-oriented care services for residents, in turn improving the residents’ quality of life.

The reported medication error rate of 0.1% was already quite low in the preimplementation phase. However, in the postimplementation phase, the Program further significantly reduced the error rate to zero. This result matched with the study of Beobide Tellería et al, which found a reduction of 91% in dispensing errors after using ATDPS in 7 OAHs in Spain [3]. Several features of the Program may have contributed to the reduced rate of medication errors. First, the orderly arrangement of electronic medication profiles in the SMMS reduces transcription errors. Second, the assistance of the automated ATDPS, together with its unique features such as using a photoelectric sensor to ensure that medications are correctly sorted into the pouches, can reduce fatigue from repetitive processes that were traditionally carried out manually, thus improving accuracy. Finally, value-added services in the hub can also help reduce errors. Pharmacists apply their professional expertise to check for medication-related problems, such as the duplication of medication therapies, incorrect dosages, potential drug-drug interactions, and inappropriate long-term use of medications, upon receipt of new prescriptions in the SMMS and medications from OAHs. The pharmacists and dispensers can also further verify the contents of the drug pouches before delivery to the OAHs to ensure the accuracy of the medications packaged by the ATDPS. This multipronged approach provides additional layers of safety nets to reduce medication errors to a minimum and improve medication safety for the residents of OAHs.

We observed a considerable quantity of wasted medication in the OAHs. This problem not only increases expenditure in the public health care system, but also poses a threat to environmental safety from a public health perspective. The extra quantity of medication also puts pressure on the storage capacity of OAHs, as space is scarce in Hong Kong. Oral solid medications accounted for a particularly large proportion of wastage. Under the centralized automated packaging service of the Program, medication wastage could thus be potentially reduced, as unused oral solid medications originally dispensed for one patient can now be packaged and supplied to another patient who requires the same medication. Our pilot data suggest that technology may help to reduce the cost associated with wastage. Future work should be dedicated to evaluating the tangible benefits of the Program.

Despite the potential benefits demonstrated in this pilot study, the implementation of the Program was not without challenges. First, the mentality and learning capacity of OAH staff members were major barriers, as the Program brought a change in their usual workflow. Nursing staff might prefer to adhere to their familiar routine. A study showed that around 40% of nursing staff reported difficulty in making changes for improvement [22]. Many OAH nursing staff are also older and might be less familiar with the technologies used in the Program. Adequate training, on-site support, sufficient run-in time, changes in management, and leadership from the senior management of the OAHs are needed to help nursing staff adapt to the new service model in the Program.

Financial sustainability is also a major challenge in continuing the Program. Without extra funding support after the end of the Program, the centralized packaging service alone would cost HK $300 (US $38.5) per resident per month. None of the participating OAHs were willing to pay for such a service out of pocket due to tight budget constraints. Specifically, we need to identify strategies to fund the Program by harnessing the actual savings from reduced medication wastage, reduced dispensing and preparation work, and the elimination of certain redundant steps in the medication management process. For example, dispensing is done in both HA pharmacies and the hub (ie, it is duplicated). To streamline the current model, we propose that medications should be provided by the HA in bulk directly to the hub instead of dispensed individually to each patient at each medical follow-up. We anticipate that in the long run, this proposed model will make more cost-effective use of human and other resources, while fully unlocking the potential savings of unused medications. We will also negotiate with relevant stakeholders (including the HA and Social Welfare Department) and policy makers to advocate a more streamlined service model.

The results of this pilot study should be interpreted in light of a few important limitations. Compared with the study of Beobide Tellería et al [3], which recruited 7 OAHs, the inclusion of only 2 OAHs in this study, both of the same type, may not fully reflect the effect of the Program on other OAHs of different natures and types. The duration of data collection was relatively short and may not have sufficiently covered the variations in time efficiency and medication errors on different days. The 2-week run-in time of implementation before data collection was apparently not sufficient for the staff of OAH 2 to familiarize themselves with the new workflow. The COVID-19 pandemic caused delays to the implementation of the Program and data collection, limiting the duration and extent of the study. However, despite the modest sample size and short observation period, the pilot data demonstrated statistically significant positive outcomes.

In the time-motion study, there may have been social desirability bias, as the OAH staff might have felt scrutinized under the camera and behaved differently from usual. To reduce the pressure felt by OAH staff during data collection, only 10-minute excerpts were recorded, which may not have fully represented the time efficiency throughout the entire process. The interoperation of medication safety outcomes may have been limited by the small number of doses and underreporting of medication errors. Especially in the preimplementation phase, the OAH staff may have felt reluctant to report errors due to already heavy workloads or fear of negative consequences. Lastly, the drug disposal reports were based on residents’ prescription data entered in the SMMS, implying that the actual quantity of medication administered to patients may not have been completely accurate.

The Program consists of combining the three distinct components of SMMS, ATDPS, and eMAR. Hence, we are unable to differentiate the effect of each individual intervention in this pilot study. However, we can potentially observe differences in outcomes among other OAHs at different implementation phases of the program. The study only focused on the measurable aspects of medication preparation and checking, which may not have fully reflected the less quantifiable effects of the Program. It also did not evaluate the effect of the Program on medication administration, as it was not appropriate to videotape residents during administration and no attempt was made to detect administration errors. However, we anticipate that the use of eMARs probably enhanced the safety and efficiency of medication administration. The impact of other features of the intervention (Table S8 in Multimedia Appendix 1) on improving the conventional medication management process should be evaluated in future studies.

### Future Work

Our future work includes recruiting a wider variety of OAHs in terms of care level, staff ratio, occupancy rate, and mode of operation. This would allow us to delineate whether the complexity of residents’ medications and the working practices of nursing staff can influence the outcomes of the Program. The unmeasurable impact of the Program would be evaluated through qualitative means, such as focus group discussions and structured interviews, to gather the perspectives of OAH staff on the user-friendliness of the Program. To capture the changes in OAH staff’s roles and responsibilities more comprehensively, we would explore how time that was relieved from medication packing and checking is redistributed to fulfil other nursing tasks during the postimplementation phase. A cost-benefit analysis would also be conducted to determine the best approach to implement this Program in a resource-limited setting. The eventual goal is to explore the prospects of adapting and applying this service model on other settings that involve complicated medication management, such as rehabilitation centers, hospices, schools for students with special needs, and other types of long-term care facilities.

### Conclusion

An innovative medication management program with IT, AT, and IoT components improved the time efficiency of medication preparation and medication safety for OAHs. With streamlining of the workflow in the future, the Program might also reduce medication wastage. Therefore, the Program is a promising solution to address the current limitations in medication management in OAHs in Hong Kong. Our future work includes validating these findings prospectively in a larger sample of OAHs over a longer time horizon. Both the tangible and intangible impact of the Program on improving patient safety, efficiency, and staff satisfaction would be explored. From a service perspective, negotiation with relevant stakeholders and policy makers would be targeted at advocating a more streamlined service model so that OAHs can achieve safer and more efficient medication management service.

## Appendix

Multimedia Appendix 1 Supplementary Materials.

## Abbreviations

ANOVA: analysis of variance
AT: automation technology
ATDPS: Automated Tablet Dispensing and Packaging System
BNF: British National Formulary
eMAR: electronic medication administration record
HA: Hospital Authority
HKPCF: Hong Kong Pharmaceutical Care Foundation Ltd
IoT: Internet of Things
IT: information technology
MAR: medication administration record
OAH: old age home
SMMS: SafeMed Medication Management System
