# Supplementary material for: Medication Management Service for Old Age Homes in Hong Kong Using Information Technology, Automation Technology, and the Internet of Things: Pre-Post Interventional Study
Source: JMIR Med Inform. 2021 Feb 10;9(2):e24280. doi: 10.2196/24280 (PMC7904397; doi:10.2196/24280)
Supplement: Multimedia Appendix 1 [file medinform_v9i2e24280_app1.docx]

SUPPLEMENTARY MATERIAL

Text S1. Ethical Considerations.

Figure S1. Flow of medications and information with the use of technologies.

Text S2. Workflow and Components of the Program.

Table S1. Number of Doses Prepared and Checked in 10 Minutes.

Table S2. Converted Minutes Required to Prepare and Check 1000 Doses.

Table S3. Results in Three-way Analysis of Variance (ANOVA).

Table S4. Generalized Equation Estimating the Number of Doses Prepared or Checked in 10 Minutes.

Table S5. Types of Medication Errors in Pre- and Post-implementation Phases.

Table S6. Medications involved in the Medication Errors.

Table S7. Medication Wastage Based on Therapeutic Classes in the British National Formulary (BNF).

Table S8. List of Features of the Intervention which Improve the Conventional Medication Management Process.

Text S1. Ethical Considerations

The Survey and Behavioural Research Ethics Committee of the Chinese University of Hong Kong (Reference number: SBRE-19-106) has granted approval for this study.

We obtained verbal consent of staff members observed in old age homes (OAHs) and the Hong Kong Pharmaceutical Care Foundation (HKPCF) was obtained prior to each data collection period. We adopted verbal consent to ensure that all data were obtained anonymously. The following points were addressed in verbal consent:

1. Participation was voluntary and implied consent to the terms and conditions of the study.
2. Participation involved being observed and videotaped the usual work on medication preparation and checking. No interruptions were made during working.
3. All data were collected anonymously. No facial appearance was videotaped during time-motion observations. No identifiers were collected on medication incident reporting forms.
4. No punishment was incurred due to errors observed and reported in the study.
5. Participants had the right of withdrawal from the study at any time.
6. All data collected were only used for research purposes and were not publicly disclosed in a fashion that would identify any specific person or organization.
7. The researcher team took precautions to preserve the confidentiality of the research data and that all reports of the research would be devoid of identifiers.

In addition to verbal consent, we also included a written statement asking for consent on the top of the medication incident reporting forms (Section 6.2). The reporting of medication incidents was voluntary and anonymous in nature.

As one of the terms and conditions in the service agreement (Section 6.4), OAHs receiving the service had agreed the collection and use of data available in SafeMed Medication Management System (SMMS), including residents’ medication records and data for medication wastage, prior to the implementation of the Integrated Old Age Home Medication Management Programme.

Figure S1. Flow of medications and information with the use of technologies.


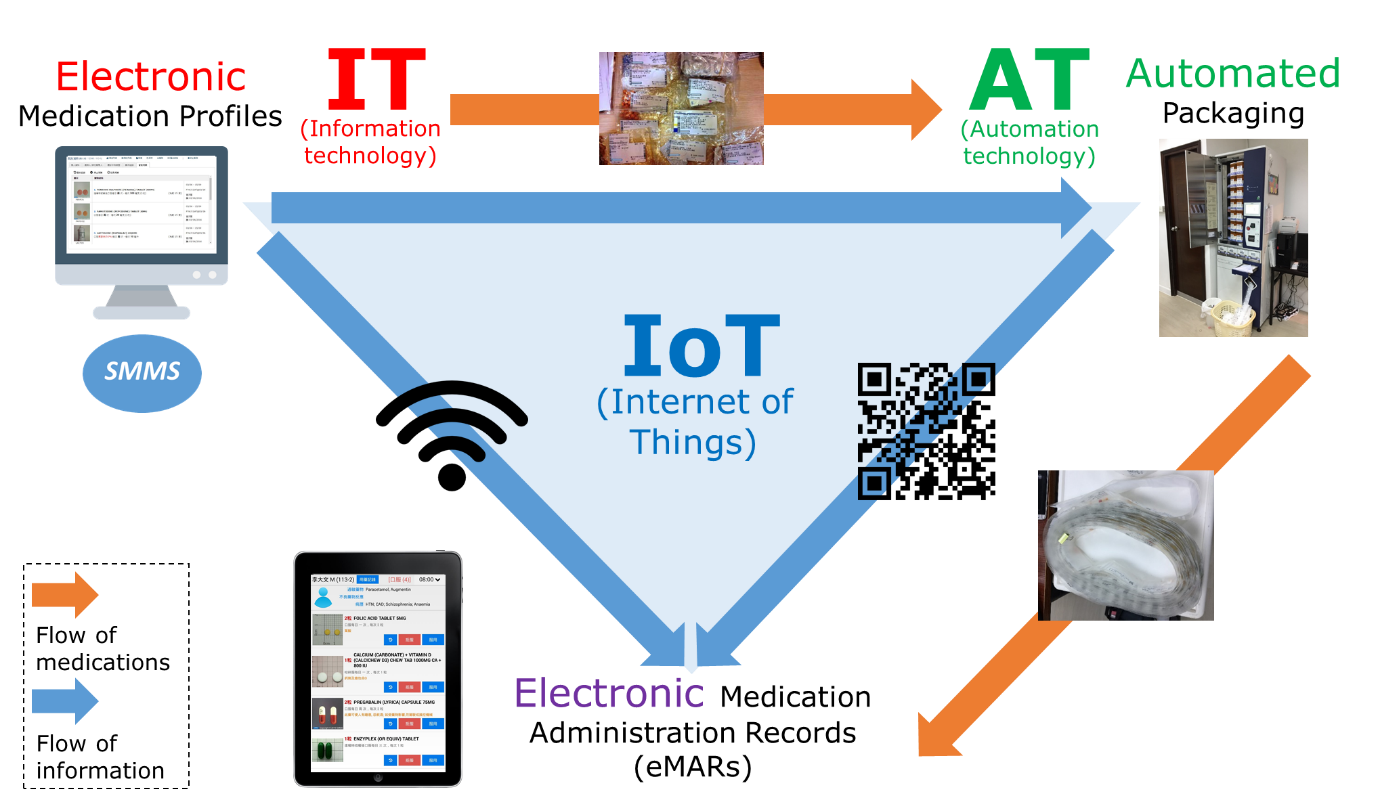


Abbreviation: SMMS, SafeMed Medication Management System.

Text S2. Workflow and Components of the Programme

1. Electronic Medication Profiles in SafeMed Medication Management System (SMMS)

Medications for elderly residents in old age homes (OAHs) are prescribed by physicians at hospitals or clinics, which are mostly the public healthcare facilities operated by Hospital Authority (HA). The medications supplied by the pharmacies of HA hospitals and clinics are either in medication plastic bags or in the original manufacturers’ packaging of medications. Labels according to the format of the HA system are stuck on the packaging of medications.

The medications are then collected by staff members from OAHs. Staff members of OAHs enter patients’ information and medications on SMMS. The electronic medication record on SMMS enables the generation of systematic medication profiles for individual patients.

Unlike other existing systems for OAHs, SMMS contains a comprehensive drug database which fully covers the medications in the formulary of HA. The code, generic name, trade name, dosage form, strength, legal classification, therapeutic class, precautions, common instructions for use, Hong Kong registration number and manufacturer are available in the drug database. Authentic photos of medications are also available for identification based on the colour, shape and markings of medications. The drug database is managed by pharmacists and updated regularly. A database of residents’ information is also included in SMMS. With the entries from OAH staff members, the personal particulars, identification photo, information of emergency contact person, medical illnesses, follow-up consultations at hospitals or clinics, admissions to hospitals and current medications of residents are stored in this database.

Based on the entries of medications from OAH staff members, verification labels are printed and stuck on the medication bags. The labels generated in SMMS provide more detailed information compared to those in the HA system. Besides the information shown on the HA label, such as patient’s Chinese name, the name, strength, quantity and code of the medication, additional information, including the Hong Kong registration number, batch number, expiry date, manufacturer and Quick Response (QR) code of the medication, is printed on the verification label generated in SMMS. Such additional information enables the tracking of medications in case a batch is found to be problematic.

1. Centralised Automated Multi-dose Packaging with Automated Tablet Dispensing and Packaging System (ATDPS)

Tablets and capsules are collected by staff members of the Hong Kong Pharmaceutical Care Foundation (HKPCF) and taken to the Centralised Dispensing Hub of HKPCF, where ATDPS is present. In the Centralised Dispensing Hub of HKPCF, medications from each OAH are placed in separated drug cabinets, and those of each patient are placed in separated drug trays. At the front of each drug tray, there is a card showing the name, OAH, assigned code, sex and age of patient, as well as a QR code specific to each patient for scanning.

With the connection of SMMS to ATDPS, medication information of patients in OAHs on SMMS is also sent to ATDPS at the Centralised Dispensing Hub of HKPCF for dispensing and packaging. With the implementation of the programme, OAH staff members no longer need to manually prepare the medications of elderly residents for each medication round. With the idea of economy of scale, it is hoped that the off-site centralised automated packaging service could help to reduce the workload of OAH staff members in medication preparation as well as to improve the efficiency of drug distribution with automated packaging.

As there is generally no pharmacist at OAHs, pharmacists of HKPCF perform their professional expertise to check for any medication-related problems, such as duplication of medication therapies, wrong dosage of medications, drug-drug interactions, and inappropriate long-term use of medications. The checking process is critical to ensure medication safety of elderly residents in OAHs.

Medication wastage could also be possibly reduced by the intervention of pharmacists during checking. Pharmacists could request the pharmacies of hospitals or clinics not to issue the medications which are in excess, or recommend them to issue refill coupons instead of the medications, so that the surplus of medications could be prevented.

Automated multi-dose packaging is done with ATDPS such that medications of a patient at a particular time of administration are packaged in the same drug pouch. Automated packaging, instead of manual packaging, is adopted to diminish human errors under the fatigue of repetitive procedures of dispensing and packaging, hence improving medication safety. Automation could also save manpower and man-time use in medication preparation, sparing more time for pharmacists of HKPCF to perform medication reviews and education activities, as well as for nurses and healthcare assistants at OAHs to provide higher-quality, patient-oriented care services for elderly residents. It is noted that the packaging is multi-dose, not unit-dose, as multi-dose packaging reduces the time of repetitive opening of drug pouches by OAH staff members.

Currently 112 drug canisters which are tailor-made for each tablet or capsule are available in ATDPS. At the front of each drug canister are two labels: one includes the drug code and a barcode to be scanned for checking during refilling; the other one includes the name, dosage form, strength, manufacturer and Hong Kong registration number of the medication. These two labels ensure that medications are refilled correctly. Two brands of the same medication are filled in different drug canisters such that product withdrawal could be done more easily in case of manufacturing incidents.

The same medications from different patients are filled into the same drug canister. This filling process means that a medication originally dispensed by a hospital or clinic to a patient may eventually be packaged and administered to another patient who is also served by the programme. It should be noted that patients’ consent must be obtained prior to such practice, so that patients could fully understand the potential benefits and risks. As elderly patients frequently require adjustments in their medications, this practice may make better use of the medications remained unused, thus reducing medication wastage.

When a drug canister becomes empty, ATDPS makes an audio announcement reminding the refilling of medications. A dispenser is responsible for the refilling of medications. Instead of pooling, bags of medication from different patients are added into drug canisters one by one to ensure that the batch number and expiry date of medications are known in each refilling. As safety measures, checking and recording of each bag of medication are done prior to refilling. Recording is done with a tablet computer, so that the records of refilling each batch of medications could be retrieved more easily.

Some tablets can be cut into halves with the half cutters in some drug canisters of ATDPS in order to cater for the various dosages of elderly residents in a flexible manner. Tablets requiring halving but not covered by the tailor-made drug canisters of ATDPS are split into halves manually by a dispenser of HKPCF.

Some medications which are not the most common ones may not be covered by the 112 tailor-made drug canisters. In such cases, ATDPS generates a location on a detachable tablet adapter (DTA) for these drugs to be dispensed and packaged. The DTA drug tray provides extra capacity for ATDPS to perform dispensing and packaging for more medications.

During manufacturing, some medications are packaged into blisters. Therefore, a deblistering machine is available in the Centralised Dispensing Hub of HKPCF for removing medications which are not moisture-sensitive from the blisters before being packaged into multi-dose drug pouches. The machine can be adjusted for removing medications of different shapes.

After the automated packaging of medications, multi-dose drug pouches are rolled with an automatic winder, which is also called a spooler, to maintain tidiness of a large number of drug pouches.

The roll of drug pouches is transferred to a drug verification machine which checks medications at a rate of approximately 1.2 seconds per drug pouch. By matching medications with their authentic photos in the database of the machine, the quantity, colour, shape and contamination of medication in each multi-dose drug pouch are examined. A judgement result list is shown on the display screen of the checking machine. The checking machine could avoid attention fatigue as in the case of manual verification. It could also save the time of manual flipping of drug pouches during checking and hence improve the efficiency of drug verification. To further ensure the accuracy of medications, the images of drug pouches on the display screen are re-verified by a pharmacist.

1. Electronic Medication Administration Records (eMARs) in SMMS

The packaged medications are delivered by staff members of HKPCF to OAHs. OAH staff members prepare, check and administer medications to patients according to the MARs generated in SMMS. The MARs comply with the requirements stated in the Guidelines on Drug Management in Residential Care Homes. They are available in paper MAR forms or eMARs.

Although both paper MAR forms and eMARs allow OAH staff members to make records upon administration of medications to elderly residents, eMARs have several advantages over paper MAR forms. First, the records of medications to be administered to patients are updated in a real-time manner. Additional modification of printed MAR forms due to updates in medication profile can be avoided. Second, only medications to be administered at a particular time are shown on eMARs during the time of drug administration. eMARs could help to reduce errors in administering medications at the wrong time. Third, eMARs are displayed on handy tablet computers. The time spent on printing paper MAR forms and flipping them during medication administration is saved when eMARs are used, so that more time could be spent on other nursing care tasks.

Moreover, eMARs aim to achieve the five rights in medication administration, including right time, right route, right patient, right drug and right dose, through multiple features. Right time is achieved by showing a list of patients with medications to be administered at the earliest medication round and reminders for any overdue drug administration. Right route is achieved by classifying medications into three groups according to routes of administration, including oral medications, non-oral medications and injections. Right patient is achieved by showing the photos of residents, in additional to their names, for identification during medication administration. Right drug and right dose are achieved by matching the medications and their strengths with their authentic photos for counter-checking.

Records of medications administered are made to prevent duplicate administration, while records of medications not administered as well as the reasons are also made for healthcare providers to understand the underlying cause of non-administration and adjust the therapeutic regimen in a patient-oriented manner. For injectable medications like insulin, injection site maps are available for recording the injection site directly on eMARs. Furthermore, as a measure of medication safety, alerts of drug allergies are popped out before drug administration in case a patient is allergic to a medication to be administered in order to prevent ADEs in patients.

Table S1. Number of Doses Prepared and Checked in 10 Minutes

| **Process** | **Phase** | **RCHE 1** | | **RCHE 2** | | **RCHE 1 & RCHE 2 combined** | |
| --- | --- | --- | --- | --- | --- | --- | --- |
|  |  | **Mean** | **SD** | **Mean** | **SD** | **Mean** | **SD** |
| Medication Preparation | Pre-implementation Phase | 13.1 | 3.7 | 35.3 | 7.7 | 24.2 | 12.8 |
|  | Post-implementation Phase | 77.1 | 9.1 | 84.8 | 15.3 | 81.0 | 12.9 |
| Medication Checking | Pre-implementation Phase | 24.7 | 12.6 | 92.0 | 7.1 | 58.4 | 35.9 |
|  | Post-implementation Phase | 67.2 | 13.0 | 53.1 | 33.7 | 60.2 | 25.9 |
| Medication Preparation & Checking combined | Pre-implementation Phase | 18.9 | 10.8 | 63.7 | 30.0 | 41.3 | 31.8 |
|  | Post-implementation Phase | 72.2 | 12.0 | 69.0 | 30.2 | 70.6 | 22.8 |

Abbreviations: RCHE, Residential Care Home for the Elderly; SD, standard deviation.

Table S2. Converted Minutes Required to Prepare and Check 1000 Doses

| **Process** | **Phase** | **RCHE 1** | | **RCHE 2** | | ***RCHE 1 & RCHE 2 combined*** | |
| --- | --- | --- | --- | --- | --- | --- | --- |
|  |  | **Mean** | **SD** | **Mean** | **SD** | **Mean** | **SD** |
| Medication Preparation | Pre-implementation Phase | 823.61 | 238.42 | 297.52 | 75.59 | 560.57 | 320.10 |
|  | Post-implementation Phase | 131.33 | 15.36 | 121.28 | 20.82 | 126.31 | 18.54 |
| Medication Checking | Pre-implementation Phase | 464.75 | 136.36 | 109.32 | 9.04 | 287.04 | 205.16 |
|  | Post-implementation Phase | 153.99 | 30.39 | 252.49 | 122.27 | 203.24 | 100.36 |

Conversion formula: 1000 ÷ (Number of Doses ÷ 10)

Abbreviations: RCHE, Residential Care Home for the Elderly; SD, standard deviation.

Table S3. Results in Three-way Analysis of Variance (ANOVA).

| Variable | p-value |
| --- | --- |
| Phase | < .001 |
| OAH | < .001 |
| Process | .057 |
| Phase stratified by OAH | < .001 |
| Phase stratified by Process | < .001 |
| OAH stratified by Process | .096 |
| Phase stratified by OAH and Process | < .001 |

Abbreviation: OAH: old age home.

Table S4. Generalized Equation Estimating the Number of Doses Prepared or Checked in 10 Minutes

| Number of doses = 35.3 + 49.5 ^(a)^ – 22.2 ^(b)^ + 14.5 ^(c)^ – 88.4 ^(d)^ + 66.9 ^(e)^ |
| --- |
| (a) 49.5 was added for post-implementation phase; |
| (b) 22.2 was subtracted for OAH 1; |
| (c) 14.5 was added for OAH 1 in post-implementation phase; |
| (d) 88.4 was subtracted for medication checking in post-implementation phase; |
| (e) 66.9 was added for medication checking in OAH 1 in post-implementation phase. |

Abbreviation: OAH, old age home.

Table S5. Types of Medication Errors in Pre- and Post-implementation Phases.

| Types of Medication Errors | Number of Doses in OAH 2 | |
| --- | --- | --- |
|  | Pre-implementation | Post-implementation |
| Missed Medication | 5 | 0 |
| Wrong Dose | 4 | 0 |
| Wrong Time of Administration | 1 | 0 |
| Total | 10 | 0 |

Abbreviation: OAH, old age home.

Table S6. Medications involved in the Medication Errors

| Medication | | Number of Doses in OAH 2 | |
| --- | --- | --- | --- |
| Therapeutic Class^a^ | Name of Medication | Pre-implementation | Post-implementation |
| Central Nervous System | Sulpiride | 2 | 0 |
|  | Memantine | 1 | 0 |
|  | Paracetamol | 1 | 0 |
|  | Sertraline | 1 | 0 |
| Cardiovascular System | Amlodipine | 2 | 0 |
|  | Aspirin | 1 | 0 |
| Blood and Nutrition | Multivitamins | 1 | 0 |
| Not applicable | Unspecified | 1 | 0 |
| Total | | 10 | 0 |

Abbreviation: OAH, Residential Care Home for the Elderly.

^a^ Based on Therapeutic Classes in British National Formulary

Table S7. Medication Wastage Based on Therapeutic Classes in British National Formulary.

| Therapeutic Classes | Cost (HK$) | |
| --- | --- | --- |
|  | OAH 1 | OAH 2 |
| 1. Gastro-Intestinal System | 504.71 | 807.08 |
| 1. Cardiovascular System | 207.16 | 1106.20 |
| 1. Respiratory System | 286.61 | 130.33 |
| 1. Central Nervous System | 1328.24 | 670.27 |
| 1. Infections | 0 | 2.60 |
| 1. Endocrine System | 76.63 | 590.33 |
| 1. Obstetrics, Gynaecology, and Urinary-Tract Disorders | 0 | 0 |
| 1. Malignant Disease and Immunosuppression | 0 | 0 |
| 1. Nutrition and Blood | 79.64 | 1613.70 |
| 1. Musculoskeletal and Joint Diseases | 0.11 | 0 |
| 1. Eye | 75.19 | 325.90 |
| 1. Ear, Nose and Oropharynx | 7.48 | 1.78 |
| 1. Skin | 0 | 0 |
| 1. Immunological Products and Vaccines | 0 | 0 |
| 1. Anaesthesia | 0.26 | 1.29 |
| Total | 2566.03 | 5249.48 |

Abbreviation: OAH, old age home.

Table S8. List of features of the intervention which improve the conventional medication management process

| Intervention features | Improvements over conventional process |
| --- | --- |
| SafeMed Medication Management System (SMMS) | |
| Electronic, structured prescription input with the help of a comprehensive drug database | - Improve efficiency over manual copying or free-text typing - Reduce errors made in manual copying or non-system free-text typing when recording prescriptions |
| Automatic summary of current medications from different prescribing sources | - Reduce errors arising from manually merging medications from different prescribing sources |
| Automated Tablet Dispensing and Packaging System (ATDPS) | |
| Automation-assisted dispensing workflow | - Improve efficiency and reduce errors due to fatigue of repetitive work in manual medication preparation |
| Dedicated checking application | |
| Photos of the medications are provided | - Improve efficiency and reduce errors as the checker does not need to rely on the actual medication or memory for the appearance of the medication |
| Medication orders are presented in an easy to understand list or matrix format | - Improve efficiency and reduce errors by reducing mental load |
| Any updates to the medication profile after packaging of medications are flagged up | - Improve accuracy of checking as paper MAR may become outdated if not manually updated in time |
| Real-time electronic signature | - Improve accountability - Reduce signature forging |
| Electronic Medication Administration Records (eMARs) | |
| Only residents with administrations due are shown, together with counters for residents completed and pending administration | - Improve efficiency and reduce chance of leaving out a resident as conventionally, the nurses need to flip through the paper MARs one by one to find out who needs medication administration at that time |
| Most up-to-date medication profile can be shown | - Improve accuracy of administration as paper MAR may become outdated if not manually updated in time |
| Counter for late administration | - Reduce chance of leaving out a resident |
| Photos of the residents and medications are provided | - Improve efficiency and reduce errors as the nurse does not need to rely on memory for identifying the resident or medications |
| Real-time electronic signature | - Improve accountability - Reduce signature forging |
